# Vaccine-induced neutralizing antibody responses to seasonal influenza virus H1N1 strains are not enhanced during subsequent pandemic H1N1 infection

**DOI:** 10.3389/fimmu.2023.1256094

**Published:** 2023-08-24

**Authors:** Petra Mooij, Daniella Mortier, Aafke Aartse, Alexandre B. Murad, Ricardo Correia, António Roldão, Paula M. Alves, Zahra Fagrouch, Dirk Eggink, Norbert Stockhofe, Othmar G. Engelhardt, Ernst J. Verschoor, Marit J. van Gils, Willy M. Bogers, Manuel J. T. Carrondo, Edmond J. Remarque, Gerrit Koopman

**Affiliations:** ^1^ Department of Virology, Biomedical Primate Research Centre, Rijswijk, Netherlands; ^2^ Department of Medical Microbiology and Infection Prevention, Laboratory of Experimental Virology, Amsterdam UMC, Location University of Amsterdam, Amsterdam, Netherlands; ^3^ Instituto de Biologia Experimental e Tecnológica (IBET), Oeiras, Portugal; ^4^ Instituto de Tecnologia Química e Biológica António Xavier, Universidade Nova de Lisboa, Oeiras, Portugal; ^5^ Infectious Diseases, Amsterdam Institute for Infection and Immunity, Amsterdam, Netherlands; ^6^ Wageningen Bioveterinary Research/Wageningen University & Research, Lelystad, Netherlands; ^7^ Vaccines, Science, Research and Innovation Group, Medicines and Healthcare Products Regulatory Agency, Hertfordshire, United Kingdom

**Keywords:** influenza, original antigenic sin, back-boosting, non-human primates, antibody response, primary response

## Abstract

The first exposure to influenza is presumed to shape the B-cell antibody repertoire, leading to preferential enhancement of the initially formed responses during subsequent exposure to viral variants. Here, we investigated whether this principle remains applicable when there are large genetic and antigenic differences between primary and secondary influenza virus antigens. Because humans usually have a complex history of influenza virus exposure, we conducted this investigation in influenza-naive cynomolgus macaques. Two groups of six macaques were immunized four times with influenza virus-like particles (VLPs) displaying either one (monovalent) or five (pentavalent) different hemagglutinin (HA) antigens derived from seasonal H1N1 (H1N1) strains. Four weeks after the final immunization, animals were challenged with pandemic H1N1 (H1N1pdm09). Although immunization resulted in robust virus-neutralizing responses to all VLP-based vaccine strains, there were no cross-neutralization responses to H1N1pdm09, and all animals became infected. No reductions in viral load in the nose or throat were detected in either vaccine group. After infection, strong virus-neutralizing responses to H1N1pdm09 were induced. However, there were no increases in virus-neutralizing titers against four of the five H1N1 vaccine strains; and only a mild increase was observed in virus-neutralizing titer against the influenza A/Texas/36/91 vaccine strain. After H1N1pdm09 infection, both vaccine groups showed higher virus-neutralizing titers against two H1N1 strains of intermediate antigenic distance between the H1N1 vaccine strains and H1N1pdm09, compared with the naive control group. Furthermore, both vaccine groups had higher HA-stem antibodies early after infection than the control group. In conclusion, immunization with VLPs displaying HA from antigenically distinct H1N1 variants increased the breadth of the immune response during subsequent H1N1pdm09 challenge, although this phenomenon was limited to intermediate antigenic variants.

## 1 Introduction

Each year, influenza virus infection causes approximately 3 to 5 million cases of severe illness and 290,000 to 650,000 deaths worldwide (World Health Organization, https://www.who.int/news-room/fact-sheets/detail/influenza-(seasonal). Current vaccination strategies generally induce short-lived immune responses of narrow specificity that are only effective against highly similar seasonal influenza virus strains (1–3); they provide minimal protection against avian influenza viruses (4). Novel vaccine strategies are under investigation to broaden responses to heterologous and heterosubtypic influenza viruses, but these strategies can be hindered by skewing phenomena related to previous exposure, described as original antigenic sin (OAS). The concept of OAS was first formulated in the mid-1950s, on the basis of studies in which antibodies induced by the initial influenza virus encounter were maintained at the highest levels during subsequent vaccinations and infections with heterologous strains (5–9). This concept has been supported by more recent longitudinal data, which show that exposure to strains encountered later in life “back-boosts” the antibody response to strains of the same subtype that were encountered earlier in life (10–13).

Importantly, the emergence of the 2009 pandemic H1N1 virus (H1N1pdm09) demonstrated that exposure to a highly divergent heterologous influenza virus strain can induce broadly cross-reactive antibodies that recognize the relatively conserved hemagglutinin (HA)-stem domain (14–16). Similar cross-reactive, HA-stem antibodies were induced after immunization with heterologous or heterosubtypic influenza viruses (17–21). Exposure to heterologous influenza virus strains also induced broadly cross-reactive antibodies that recognized other conserved epitopes in the head domain or trimeric interface (22–24). It is still unclear whether exposure to H1N1pdm09 also enhances responses to previously encountered pre-pandemic seasonal H1N1 (H1N1) influenza virus strains.

Studies in ferrets have demonstrated that sequential infection with heterologous H1N1 influenza virus strains can induce cross-reactive antibodies that recognize H1N1pdm09 and provide partial protection against H1N1pdm09 infection (25). Sequential immunization with HA from heterologous H1N1 strains also led to the induction of HA-stem antibodies in mice, ferrets, and non-human primates (26, 27). Similar broadening of the immune response against influenza was induced in mice and ferrets by multivalent vaccine approaches that consist of a mixture of HAs from dissimilar influenza strains (28–30). These results appear to contradict the low HA-stem and low virus-neutralizing responses, as well as the lack of protection, when H1N1pdm09 emerged in the human population (16, 31, 32). Strikingly, individuals aged >50 years were less susceptible (32), presumably because of previous exposure to H1N1 viruses with greater similarity to H1N1pdm09, as suggested by serological studies using the 1976 swine influenza vaccine (33, 34). Because influenza virus exposure and/or vaccination histories in humans are complex and partially unknown, it is difficult to fully determine the conditions in which previous exposure to H1N1 influenza can lead to cross-protection against H1N1pdm09.

In this study, we explored these issues using non-human primates as a naive animal model with high homology to humans in terms of immune system components (35–37). Animals were immunized with VLPs displaying HA of influenza virus A/Brisbane/59/2007 (H1N1) alone (monovalent vaccine) or with VLPs displaying a combination of five different seasonal HAs covering >50 years of viral evolution (pentavalent vaccine), then challenged with A/California/04/2009 (H1N1pdm09) influenza virus. Neither vaccine strategy induced any cross-neutralizing antibody responses to H1N1pdm09 or measurable HA-stem responses, and both strategies did not protect against H1N1pdm09 infection; however, enhanced induction of anti-stem responses and antigen-dependent phagocytosis (ADP) were observed after H1N1pdm09 challenge. Furthermore, there was an increase in the breadth of virus neutralization responses to antigenically intermediate influenza virus A/swine/Iowa/15/30 and A/New Jersey/8/76 H1N1 strains, but there was no back-boosting of responses to the original H1N1 vaccine strains.

## 2 Materials and methods

### 2.1 Animals

This study was performed using 16 outbred, mature, male cynomolgus macaques (*Macaca fascicularis)*. Animals were bred in captivity for research purposes and socially housed in ABSL-III facilities at the Biomedical Primate Research Centre, Rijswijk, The Netherlands (an AAALAC-accredited institution). Non-human primate housing and care followed international guidelines (The European Council Directive 86/609/EEC, Convention ETS 123 with revised Appendix A, and the ‘Standard for Humane Care and Use of Laboratory Animals by Foreign Institutions’ [United States National Institutes of Health]). All animal handling was performed within the Department of Animal Science, in accordance with Dutch law. Animals tested negative for antibodies that recognized simian type D retrovirus and simian T-cell lymphotropic virus; they were screened for the absence of antibodies that recognized influenza A/Puerto Rico/8/34 (H1N1) viral lysate (Advanced Biotechnologies Inc., Eldersburg, MD, USA). All animals were classified as healthy based on physical examination and assessments of complete blood count and serum chemistry.

Animals were housed in pairs with socially compatible cage mates and maintained on a 12-hour light/dark cycle. They were offered a daily diet consisting of monkey food pellets, fruit, and vegetables. Daily enrichment was provided in the form of pieces of wood, mirrors, food puzzles, and various other homemade or commercially available enrichment products. Drinking water was provided *ad libitum* through an automatic watering system. Veterinary staff performed daily health checks before infection; they recorded appetite, general behavior, and stool consistency. During the influenza virus infection experiment, animals were checked twice daily; clinical symptoms (skin and fur abnormalities, posture, eye and nasal discharge, sneezing and coughing, and respiratory rate) were scored using a previously published system (38). A score of ≥35 was regarded as a predetermined endpoint and justification for euthanasia. Animals were weighed each time they were sedated. Hematology parameters were measured in a Sysmex XT-2000iV Automated Hematology Analyzer (Sysmex^®^ Corporation of America) using blood that had been mixed with ethylenediaminetetraacetic acid to prevent coagulation.

The Institutional Animal Care and Use Committee of the Biomedical Primate Research Centre (dierexperimentencommissie, DEC-BPRC) approved the study protocol (DEC715C), which was developed in accordance with strict international ethical and scientific standards and guidelines. The qualifications of the members of this committee, including their independence from the research institute, followed Dutch law regarding animal experiments (Wet op de Dierproeven, 1996).

### 2.2 Study design and influenza virus detection

**Figure 1:**
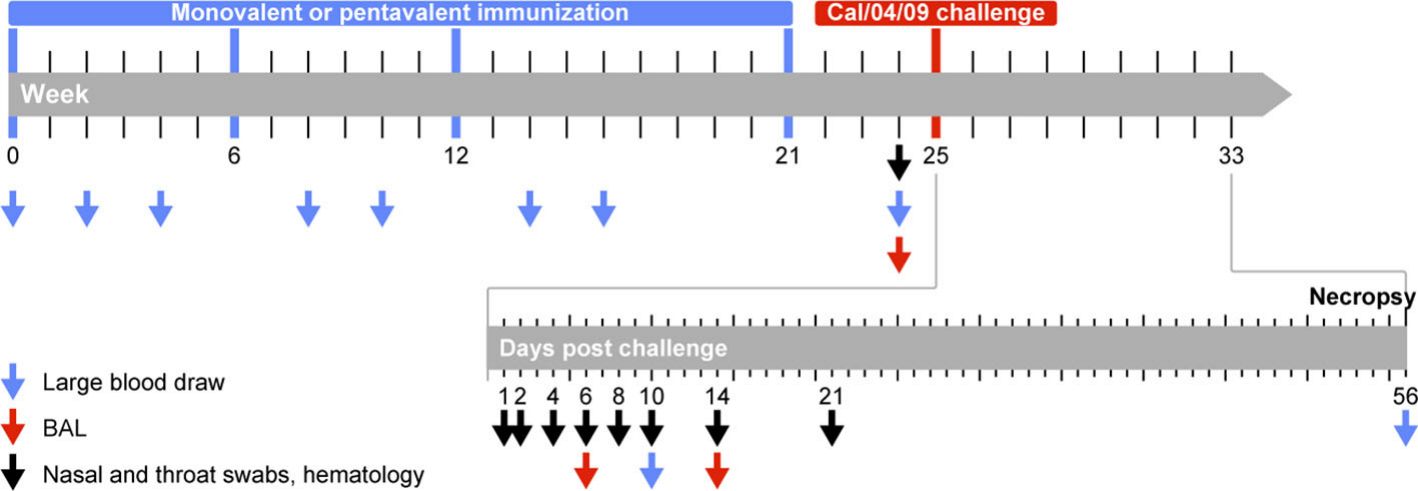
Study protocol. Animals were immunized in weeks 0, 6, 12, and 21 (indicated by blue bar) with VLPs displaying HA of seasonal influenza A (H1N1) virus strains. Animals received monovalent VLPs displaying HA from a single HA variant (influenza A/Brisbane/59/2007; monovalent vaccine group) or pentavalent VLPs displaying a combination of HA from five different variants: A/Puerto Rico/8/34 (PR8/34), A/USSR/92/77, A/Texas/36/91, A/New Caledonian/20/99 (NC/20/99), A/Brisbane/59/07 (Bris/59/07) (pentavalent vaccine group). Antibody responses were measured 4 weeks after the first, second, and third immunization procedures (weeks 4, 10, and 16) and 3 weeks after the fourth immunization procedure (week 24). T cell responses were measured 2 weeks after the first, second, and third immunization procedures (weeks 2, 8, and 14). All immunized animals and 4 naive control animals were challenged (indicated in red) in week 25 with pandemic influenza A/California/04/2009 virus (Cal/04/09). Blood, nasal swabs, and throat swabs were collected on days 1, 2, 4, 6, 8, 10, 14, and 21 after infection; BAL fluid was collected on days 6 and 14 to monitor virus replication.

Animals were randomly assigned to three groups, using the “aselect” function in the Excel program (Microsoft) to generate random numbers; animals with the lowest numbers were assigned to the monovalent vaccine group, animals with intermediate numbers were assigned to the pentavalent vaccine group, and animals with the highest numbers were assigned to the control group. Ages and weights were similar between the groups (**Table S1**). One group of six animals was immunized with monovalent VLPs displaying HA from the seasonal influenza virus strain A/Brisbane/59/2007 (H1N1) (monovalent vaccine group). A second group of six animals was immunized with pentavalent VLPs displaying a combination of five different seasonal influenza virus-HAs: influenza A/Puerto Rico/8/34 (H1N1), influenza A/USSR/92/77 (H1N1), influenza A/Texas/36/91 (H1N1), influenza A/New Caledonia/20/99 (H1N1), and influenza A/Brisbane/59/2007 (H1N1) (pentavalent vaccine group). Influenza M1 from strain A/California/04/2009 was used as a scaffold. All VLPs were produced in insect High Five cells using the baculovirus expression vector system, then purified using a combination of filtration and chromatography techniques described elsewhere (39, 40). A third naive control group of four animals received injections of NaCl. Immunization was performed by intramuscular delivery at a single site on the upper left arm in weeks 0, 6, 12, and 21 (**Figure 1**). Animals in the monovalent and pentavalent vaccine groups received VLPs with a total HA content of 15 μg during the first three immunization procedures. Because no cross-neutralizing responses had been induced against the challenge virus the animals received VLPs with an increased total HA content of 24 μg during the fourth immunization procedure. Local reactions (e.g., edema, redness, and induration) were recorded on days 1, 2, and 3 after each immunization procedure.

Four weeks after the last immunization procedure, all 16 animals were challenged with a suspension containing 10^6^ TCID_50_/mL influenza A/California/04/2009 (H1N1pdm09) virus (Cal/04/09) by intrabronchial (2 mL left lung, 2 mL right lung, with a bronchoscope), intranasal (0.5 mL per nostril), oral (1 mL), and conjunctival (0.1 mL per eyelid) administration; the total amount of virus was 6.2x10^6^ TCID_50_. A TCID_50_ assay on Madin-Darby canine kidney (MDCK) cells revealed that the titer of the viral stock was 10^7.07^ (11,748,976) TCID_50_/mL.

Tracheal and nasal swabs were collected before and 1, 2, 4, 6, 8, 10, 14, and 21 days after challenge using Copan flocked swabs (FLOQswabs, 502CS01, Copan, Italy) (**Figure 1**). Bronchoalveolar lavage (BAL) fluid was collected on days 6 and 14 after infection using a bronchoscope. Viral RNA was isolated using a QIAamp Viral RNA Mini kit (Qiagen Benelux BV, Venlo, The Netherlands) in accordance with the manufacturer’s instructions; it was detected by real-time polymerase chain reaction, as previously described (41).

### 2.3 Humoral immune responses

Serum samples were tested for the presence of anti-influenza virus antibodies by enzyme-linked immunosorbent assays (ELISAs), using plates coated with 1 μg/mL of influenza antigen A/California/07/2009 (Pandemrix, GlaxoSmithKline, London, United Kingdom), or influenza antigens A/PR/8/34, A/USSR/92/77, A/Texas/36/91, A/New Caledonia/20/99, and A/Brisbane/59/2007 (whole viruses were grown in eggs, inactivated, and purified by zonal centrifugation, Zydus Cadila, Ahmedabad, Gujarat, India), as previously described (42). The trimeric HA-stem and monomeric HA-head domain proteins from A/Netherlands/602/2009 (H1N1pdm09) were produced as previously described and used to coat Ni-NTA plates for ELISA-based antibody detection (43). Hemagglutination inhibition (HAI) and microneutralization assays were performed on serum samples as previously described (42). To remove non-specific agglutination inhibitors, serum samples were treated with receptor-destroying enzyme and incubated for 1 hour with 0.25 % turkey red blood cells in phosphate-buffered saline (PBS). Viruses tested in these assays were: influenza A/California/04/2009 (Medicines and Healthcare products Regulatory Agency [MHRA], Hertfordshire, United Kingdom), A/PR/8/34 (MHRA), A/Brisbane/59/2007 (MHRA), A/USSR/92/77 (MHRA), A/Texas/36/91 (MHRA), A/New Caledonia/20/99 (MHRA), A/New Jersey/8/76 (MHRA), and NIBRG-196 (MHRA; a reassortant created by reverse genetics using HA and NA from A/swine/Iowa/15/30 and all other genes from A/PR/8/34). Fresh stocks of all viruses were grown in MDCK cells in accordance with standard procedures; viral titers were determined using a TCID_50_ assay on MDCK cells.

### 2.4 ADP and antibody-dependent cellular cytotoxicity (ADCC)

HA-specific ADP assays were performed as previously described (44–46). Briefly, yellow-green–labeled NeutrAvidin FluoSpheres (1 μm; Invitrogen) were coated with biotinylated recombinant HA from influenza A/California/07/2009 (H1N1) (Sino Biological Inc., Beijing, China), incubated with serially diluted serum samples, then cultured with THP-1 cells for 16 hours at 37°C. Phagocytosis was measured by fluorescence-activated cell sorting using an LSRII flow cytometer (BD Biosciences, San Jose, CA, USA). ADCC activity was measured using a plate-bound natural killer (NK)-cell activation assay, as previously described (47, 48). The wells of a 96-well ELISA plate (Maxisorp, Nunc) were coated overnight at 4°C with 400 ng/well purified HA protein (influenza A/California/07/2009 (H1N1), Sino Biological Inc.) in PBS. Wells were washed three times with PBS, then incubated with heat-inactivated serum (56°C for 60 minutes), diluted in a 1% bovine serum albumin in PBS solution, for 2 hours at 37°C. Subsequently, serum was discarded and 0.5x10^6^ thawed peripheral blood mononuclear cells (PBMCs) from a healthy macaque, suspended in R10 medium (RPMI supplemented with 10% fetal calf serum, penicillin, streptomycin, and L-glutamine; Gibco, Life Technologies), were added to each well. Additionally, anti-human CD107a^APC^ antibody (H4A3 clone; BD Biosciences), 5 mg/mL brefeldin A (GolgiPlug, BD Biosciences), and 5 mg/ml monensin (Golgi Stop; BD Biosciences) were added and incubated in 5% CO_2_ for 5 hours at 37°C. Next, cells were stained with LIVE/DEAD^®^ Fixable Violet dead cell stain (Molecular Probes), then incubated with CD3^FITC^ (clone SP34, BD Biosciences), CD14^PE-TexasRed^ (clone RMO52, Beckman Coulter), and NKG2a^PE^ (clone Z199; Beckman Coulter) for 30 minutes at room temperature in the dark. Cells were fixed with 2% paraformaldehyde overnight at 4°C, and data were acquired using an LSRII flow cytometer (BD Biosciences).

### 2.5 Interferon-gamma (IFNγ) ELISpot assay

IFNγ ELISpot assays were performed in triplicate, in accordance with the manufacturer’s protocol (U-CyTech biosciences, Utrecht, The Netherlands) and as previously described (42). Briefly, 1.2x10^6^ freshly isolated PBMCs were stimulated for 16 hours with influenza A/Brisbane/59/2007 (H1N1) or A/California/04/2009 (H1N1pdm09) (multiplicity of infection: 1). After stimulation, non-adherent cells were collected and plated (2x10^5^ cells/well) in polyvinylidene fluoride ELISpot plates (Millipore, Burlington, MA, USA) (with Bris/59/07 or Cal/07/04 virus stimulation) that had previously been coated with anti-IFNγ monoclonal antibody MD-1 (U-CyTech). Culture medium was used as a negative control condition; a phorbol myristate acetate/ionomycin mixture (Merck, Darmstadt, Germany) was used as a positive control condition.

### 2.6 Statistical analysis

Differences between groups were compared using the Mann–Whitney test. Differences in responses before and after challenge were determined using the paired Wilcoxon test. Two-sided p-values <0.05 were considered statistically significant.

## 3 Results

### 3.1 Immunization with monovalent and pentavalent VLPs induces strong antibody and virus-neutralizing responses to vaccine strains, but not to H1N1pdm09

**Figure 2:**
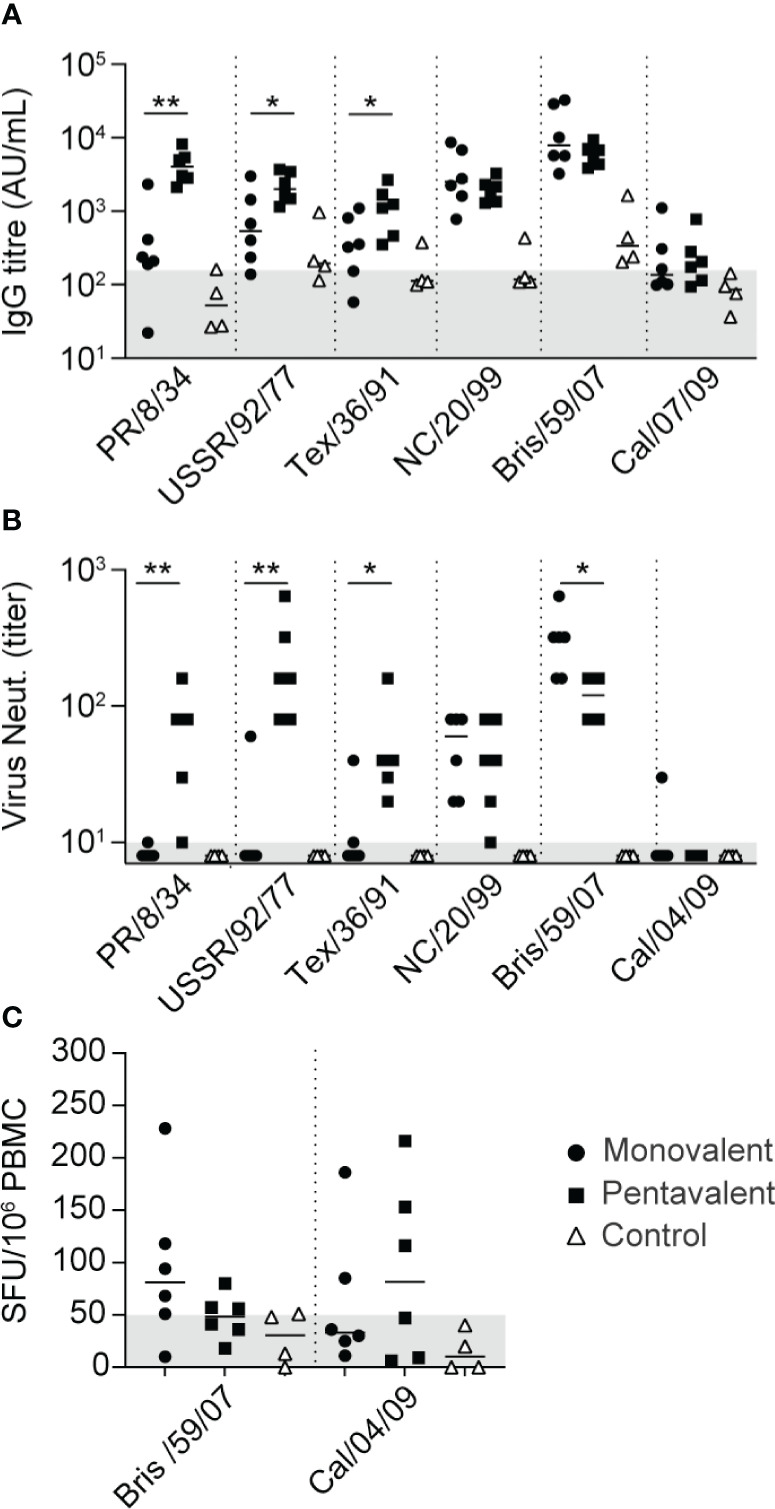
Vaccine-induced antibody and T cell immune responses. **(A)** IgG antibody responses 3 weeks after the fourth immunization procedure—measured using whole virus antigens of PR/8/34, USSR/92/77, Texas/36/91, NC/20/99, Bris/59/07, and Cal/07/09—among animals in the monovalent vaccine group (black circles), pentavalent vaccine group (black squares), and naive control group (white triangles). Antibody levels are expressed as arbitrary units (AU), defined as the dilution where the OD_450_ value is 1 unit above background. Gray area indicates the average response observed in pre-immunization serum samples (157 AU). **(B)** Neutralizing antibody titers measured 3 weeks after the fourth immunization procedure against PR/8/34, USSR/92/77, Texas/36/91, NC/20/99, Bris/59/07, and Cal/04/09 virus. Gray area indicates lowest dilution tested. **(C)** IFNγ ELISpot responses measured 2 weeks after the third immunization procedure. Cells were stimulated with Bris/59/07 or Cal/04/09 virus. Numbers of spot-forming units/10^6^ PBMCs are depicted after subtraction of background, calculated as the mean number of spots in unstimulated wells plus two times the standard deviation. Gray area indicates the threshold of responses observed in pre-immunization samples. Significant differences between immunization groups were determined using the Mann–Whitney test. *p<0.05; **p<0.01.

To test the hypothesis that immunization with multivalent H1N1 vaccine strains can expand immune responses to highly divergent strains such as H1N1pdm09, two groups of six cynomolgus macaques were immunized with VLPs expressing HA of influenza A/Brisbane/59/2007 (H1N1) alone (monovalent) or with VLPs expressing five different seasonal HAs (pentavalent). The pentavalent vaccine consisted of influenza A/PR/8/34, A/USSR/92/77, A/Texas/36/91, A/New Caledonia/20/99 (NC/20/99), and A/Brisbane/59/2007 (Bris/59/07) HAs, covering >50 years of viral evolution. The vaccine was well-tolerated; no local reactions were observed except for mild but transient local erythema. In the monovalent vaccine group, antibody responses to Bris/59/2007 and NC/20/99 were induced after the first immunization procedure (**Figure 2A** and **Figure S1**); the titers were further enhanced during the subsequent booster immunization procedure, then remained high (**Figure S1**). In this group, some weak cross-reactive responses against other H1N1 strains were detected after booster immunization procedures in a few animals. Virus-neutralizing responses to Bris/59/07 and NC/20/99 were induced, but responses to other H1N1 strains were not observed, except for a response to USSR/92/77 in one animal and a response to Tex/36/91 in another animal (**Figure 2B**). The robust responses to NC/20/99, which was not included as a vaccine strain in the monovalent vaccine group, might have been caused by its high genetic similarity to Bris/59/07 (49) (https://www.bprc.nl/sites/default/files/pubs/H1-Full-3D-Year.html). Animals that received the pentavalent vaccine developed antibody-binding and virus-neutralizing responses to all five vaccine strains (**Figures 2A, B**, and **Figure S1**). However, in the monovalent and pentavalent vaccine groups, only very low antibody-binding and no cross-neutralizing responses to Cal/04/09 (H1N1pdm09) were detected, with the exception of a low response in one animal in the monovalent vaccine group (**Figures 2A, B** and **Figure S1**). The virus-neutralizing antibody titer against Bris/59/07 was significantly lower in the pentavalent vaccine group than in the monovalent vaccine group. This may be related to the difference in vaccine dose administered; in the pentavalent vaccine group, the total dose of 15 μg HA was divided among the five antigens. HAI responses to PR/8/34, Bris/59/07, and Cal/04/09 were consistent with the virus neutralization data, revealing inhibition of only Bris/59/07 in the monovalent vaccine group, inhibition of PR/8/34 and Bris/59/07 in the pentavalent vaccine group, and low or no inhibition of Cal/04/09 in either group (**Figure S2**). Only weak cellular immune responses, as measured by IFNγ ELISpot assays, to Bris/59/07 were detected in the monovalent and pentavalent vaccine groups (**Figure 2C**). Similar weak cross-reactive responses to H1N1pdm09 virus were also detected (**Figure 2C**).

### 3.2 Immunization with monovalent and pentavalent VLPs does not protect against H1N1pdm09 infection

**Figure 3:**
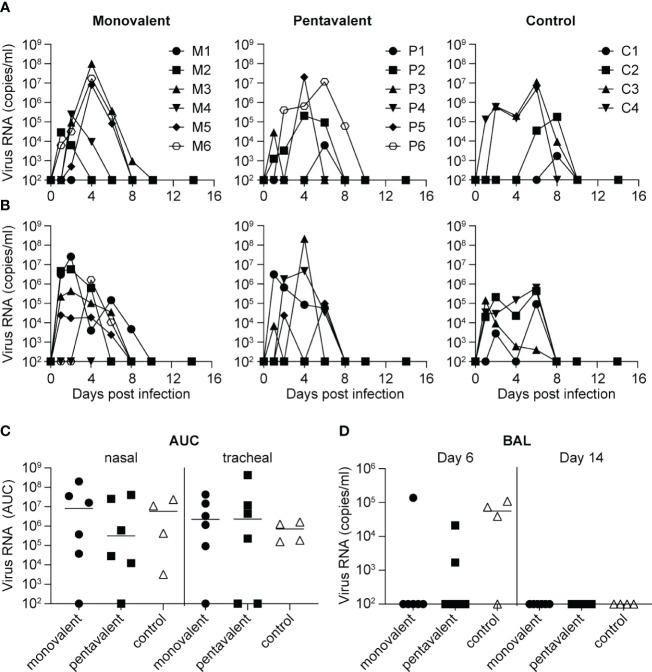
Influenza virus load after challenge with influenza A/California/04/2009 (H1N1pdm09). Viral load as determined by quantitative reverse transcription polymerase chain reaction is shown over time for each individual animal in the monovalent vaccine group (M1-M6), pentavalent vaccine group (P1-P6), and naive control group (C1-C4), based on analysis of **(A)** nasal swabs and **(B)** tracheal swabs. **(C)** Total virus production over time calculated as area under the curve (AUC) in nasal and tracheal swabs from the monovalent vaccine group (black circles), pentavalent vaccine group (black squares), and naive control group (white triangles). **(D)** Viral load detected in BAL fluid collected on days 6 and 14 after virus inoculation. Viral RNA quantity is expressed in copies/ml. There were no significant differences between groups (Mann–Whitney test).

Four weeks after the last immunization procedure, animals were challenged with a dose of 6.2 x10^6^ TCID_50_ Cal/04/09 influenza virus, administered by an intrabronchial, oral, nasal, and intraocular route. All animals in the monovalent, pentavalent, and control groups were infected (**Figure 3**). Virus was detected in nasal swabs from five of the monovalent group animals (M2, M3, M4, M5, and M6), five of the pentavalent group animals (P1, P2, P3, P5, and P6), and all four control group animals (**Figure 3A**); it was detected in throat swabs from five of the monovalent group animals (M1, M2, M3, M5, and M6), four of the pentavalent group animals (P1, P3, P4, and P5), and all four control group animals (**Figure 3B**). There were no significant differences among groups in total virus production over time, as measured by the area under the curve (**Figure 3C**). On day 6 after challenge, virus was detected in BAL fluid from one animal (M5) in the monovalent vaccine group, two animals in the pentavalent vaccine group (P4 and P6), and three animals in the control group (C1, C2, and C4). While it would have been logical to find fewer virus-positive animals in the pentavalent vaccine group than in the monovalent vaccine group it must be noted that the small differences observed here likely fall within the experimental variation that could occur in this type of experiment. There were no significant differences in BAL fluid viral load among groups (Mann–Whitney test).

### 3.3 Infection with H1N1pdm09 results in partial broadening of the immune response and enhanced HA-stem responses in immunized animals but does not cause back-boosting to H1N1 vaccine strains

**Figure 4:**
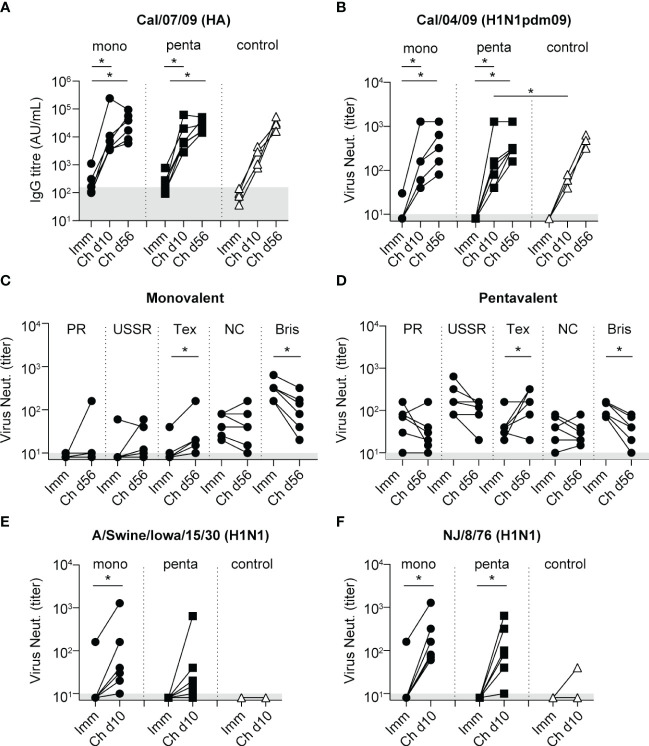
Antibody responses after challenge with influenza A/California/04/09 (H1N1pdm09). In all graphs, the antibody levels measured 3 weeks after the fourth immunization procedure are included for comparison (Imm). **(A)** IgG antibody responses to HA protein of Cal/07/09 measured 10 and 56 days after challenge (Ch d10 and Ch d56, respectively) among animals in the monovalent vaccine group (black circles), pentavalent vaccine group (black squares), and naive control group (white triangles). Antibody levels are expressed as arbitrary units (AU), defined as the dilution where the OD_450_ value is 1 unit above background. Gray area indicates the average response observed in pre-immunization serum samples (157 AU). **(B)** Neutralizing antibody titers measured 10 and 56 days after challenge against Cal/04/09 virus. Symbols as indicated for panel **(A)**. Neutralizing antibody titers measured against H1N1 vaccine strains PR/8/34, USSR/92/77, Texas/36/91, NC/20/99, and Bris/59/07 on day 56 after challenge among animals in the monovalent vaccine group **(C)** and pentavalent vaccine group **(D)**. **(E)** Neutralizing antibody titers 10 days after challenge measured against influenza A/Swine/Iowa/15/30 (H1N1). **(F)** Neutralizing antibody titers 10 days after challenge measured against influenza A/New Jersey/8/76 (H1N1). Gray area in graphs **(B–F)** indicates lowest dilution tested. Significant differences between groups were determined using the Mann–Whitney test. Significant differences between pre- and post-challenge responses were determined using the paired Wilcoxon test. *p<0.05.

After challenge with H1N1pdm09 influenza virus, there were strong increases in antibody-binding titers and virus neutralization responses to Cal/04/09; these increases began on day 10 after infection and continued until day 56 (**Figures 4A, B**, and **Figure S1**). On day 10, virus-neutralizing titers against Cal/04/09 were significantly higher in the pentavalent vaccine group than in the control group; the difference between the monovalent vaccine group and the control group was not statistically significant (**Figure 4B**). On day 56, virus-neutralizing titers had increased to a similar level in all groups (**Figure 4B**), indicating that the vaccine-mediated enhancement in the pentavalent vaccine group primarily involved a rapid rise in antibody titer.

Analysis of post-challenge responses showed that virus-neutralizing titers against the H1N1 vaccine strains PR/8/34, USSR/90/77, and NC/20/99 did not increase in the monovalent vaccine group or the pentavalent vaccine group; there were even decreases in titers against Bris/59/07 (**Figures 4C, D**). An increase in titer was observed only against Tex/36/91 (**Figures 4C, D**). HAI response results were similar; after H1N1pdm09 challenge, these responses decreased for the PR/8/34 and Bris/59/07 H1N1 strains, whereas they increased for Cal/04/09 (**Figure S2**). Antibody-binding titers increased against all H1N1 vaccine strains (**Figure S1**), but these responses may not necessarily be directed toward HA of the H1N1 strains, the ELISA coating used egg-grown inactivated whole virus preparations, implying that after challenge also responses against other viral proteins could be detected. Consistent with this assumption, the control group showed no cross-neutralizing responses to any H1N1 vaccine strains after challenge (data not shown). Because the vaccine groups appeared to lack back-boosting of neutralizing responses to H1N1 vaccine strains, which are genetically very distant from H1N1pdm09 (49), we investigated responses to two genetically intermediate strains A/swine/Iowa/15/30 (H1N1) and A/New Jersey/8/76 (NJ/08/76)(H1N1) (49). Immunization did not induce any virus-neutralizing responses to these viruses, except in one animal in the monovalent vaccine group (**Figures 4E, F**). After infection, responses to A/Swine/Iowa/15/30 (H1N1) and NJ/8/76 were both significantly increased in the monovalent and pentavalent vaccine groups; these responses were largely absent in the control group (**Figures 4E, D**). Thus, although the broadening of the immune response occurred, it was limited to strains that are genetically intermediate between H1N1pdm09 and H1N1 viruses (https://www.bprc.nl/sites/default/files/pubs/H1-Full-3D-Year.html).

**Figure 5:**
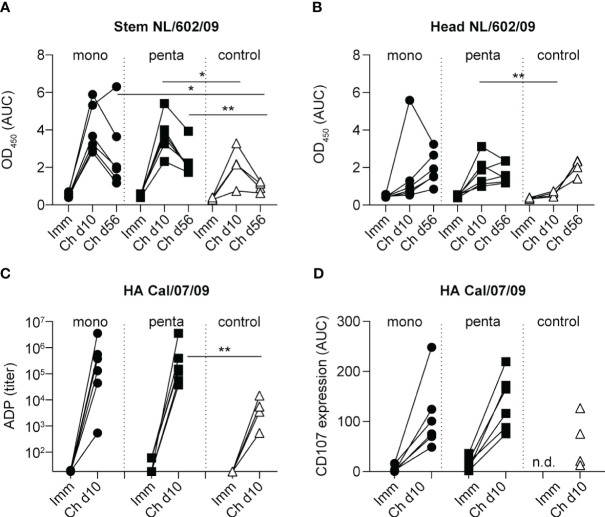
NL/602/09 (H1N1pdm09) stem and head domain specificity and ADP and ADCC responses. IgG antibody responses to **(A)** the stem domain and **(B)** the head domain of NL/602/09 HA measured 3 weeks after the fourth immunization procedure and 10 and 56 days after challenge among animals in the monovalent vaccine group (black circles), pentavalent vaccine group (black squares), and naive control group (white triangles). Antibody levels were calculated as the AUC of OD_450_ values measured in a range of serum dilutions. **(C)** ADP responses measured 3 weeks after the fourth immunization procedure and 10 days after challenge using beads coated with HA protein of Cal/07/09. The lowest dilution tested was 1:20. Symbols as indicated for panel **(A)**. **(D)** ADCC responses measured 3 weeks after the fourth immunization procedure and 10 days after challenge against the HA protein of Cal/07/09. The panel shows expression of CD107 in stimulated NK cells, calculated as the AUC of values measured in a range of serum dilutions. Symbols as indicated for panel **(A)** Significant differences between groups were determined using the Mann–Whitney test. *p<0.05; **p<0.01.

To determine whether heterologous challenge with H1N1pdm09 could induce responses to the HA-stem domain, as observed in humans (16, 20), serum samples were tested in an ELISA for binding to the NL/602/09 (H1N1pdm09) stem domain, which was compared with binding to the NL/602/09 head domain (**Figures 5A, B**). Although neither monovalent or pentavalent vaccine immunization induced antibodies to the HA-stem or HA-head of NL/602/09, there was a strong increase in HA-stem binding by day 10 after infection, which subsequently decreased by day 56. A similar transient response was previously observed in animals infected with influenza A/Mexico/InDRE4487/2009 (H1N1pdm09) (50). Similarly, responses in the control group peaked on day 10 and then declined (**Figure 5A**). The HA-stem response measured on day 56 post-infection was significantly higher in both vaccine groups, compared with the control group; this finding is consistent with observations in humans. However, on day 10 post-infection, a significant difference compared with the control group was only observed in the pentavalent vaccine group; in the monovalent vaccine group, this difference was not statistically significant (**Figure 5A**). Responses to the NL/602/09 head domain were only gradually induced, with slightly more rapid induction in the pentavalent vaccine group (**Figure 5B**), consistent with the post-challenge virus-neutralizing responses discussed above (**Figure 4B**).

Because HA-stem responses are known to support Fc receptor-mediated effector function, we investigated ADP and ADCC in pre- and post-challenge serum samples. After immunization, only very low ADP titers and CD107 induction in NK cells [a surrogate assay for NK cell effector function (48)] were observed (**Figures 5C, D**). By day 10 after infection, there were strong increases in ADP titers and CD107 induction in the monovalent and pentavalent vaccine groups, as well as the control group (**Figures 5C, D**). ADP responses in the pentavalent vaccine group, but not the monovalent vaccine group, were significantly higher than responses in the control group (**Figure 5C**), similar to the findings regarding HA-stem responses (**Figure 5A**). There were no significant differences among groups in terms of CD107 induction on NK cells (**Figure 5D**). In conclusion, the pentavalent vaccine group demonstrated accelerated induction of HA-stem and ADP responses during infection with heterologous H1N1pdm09 virus.

## 4 Discussion

The main challenge in influenza vaccine development is the induction of broadly cross-reactive immune responses that can protect human populations against emerging divergent influenza virus strains or zoonotic influenza viruses (51). Studies in mice and ferrets have shown that such broadening can be achieved by sequential infection with heterologous influenza virus strains or by simultaneous exposure to multivalent influenza virus vaccines (25, 27–30). Nonetheless, no pre-existing cross-neutralizing antibodies to H1N1pdm09 virus or protection against infection were observed in humans during the H1N1pdm09 pandemic, despite the presence of pre-existing immunity to several H1N1 strains (31, 32). To gain better insight into the role of previous exposure to H1N1 strains in generating cross-recognition of H1N1pdm09, we used non-human primates as a naive animal model closely related to humans. Similar to previous findings in humans, we did not observe induction of neutralizing or anti-Cal/04/09 HA-stem antibodies after immunization with a monovalent or pentavalent H1N1 vaccine; notably, the pentavalent vaccine covered >50 years of H1N1 viral evolution.

The results of previous studies in mice and ferrets suggested that the induction of cross-protective responses to H1N1pdm09 or HA-stem responses requires sequential infection with different H1N1 virus strains (25, 52). In contrast, another study involving mice, ferrets, and non-human primates showed that anti-H1 stem responses could also be generated by vaccination (26). Furthermore, multivalent vaccine approaches can induce broadly cross-protective immune responses to several other influenza A subtypes (28–30). Although the multivalent vaccine approach tested in the present study induced robust neutralizing antibody responses to all vaccine components, these responses did not lead to the induction of H1N1pdm09 cross-neutralizing or HA-stem responses. Nevertheless, a mild vaccine effect was demonstrated: the pentavalent vaccine group showed a higher virus neutralization titer against H1N1pdm09, higher antibody-binding titers to the HA-head and HA-stem stem domain, and a higher ADP titer on day 10 after challenge, compared with the naive control group (**Figures 4B**, **5A–C**). Therefore, the pentavalent vaccine strategy may have led to more rapid increases in potentially protective responses to H1N1pdm09. Although there were no significant differences among groups in the total amount of virus produced over time in the nose or throat, a notable finding was that virus could be detected in BAL fluid from only one of six animals in the monovalent vaccine group and two of six animals in the pentavalent vaccine group, compared with three of four animals in the control group (**Figure 3**). HA-stem antibodies can modulate several Fc receptor-mediated effector functions (53–56), which are important for the *in vivo* protective effects of these antibodies (57, 58). The HA-stem response observed in this study was accompanied by similar increases in ADP and ADCC. These HA-stem responses could potentially support viral clearance by alveolar macrophages through the ADP mechanism (59).

We observed a striking rapid induction of HA-stem responses that peaked on day 10 after infection and then declined, whereas HA-head responses appeared more slowly (**Figures 5A, B**). A similar HA-stem response phenomenon (rapid peak and decline) after H1N1pdm09 infection in naive macaques was recently described (50). In the present study, the early peak in HA-stem responses was more pronounced in H1N1-immunized animals than in the control group. Future studies should investigate whether other aspects of the B-cell immune response, such as memory B-cell formation, are similarly affected.

Except for the more rapid increases in anti-H1N1pdm09 neutralizing, head-binding, and stem-binding responses, the monovalent and pentavalent vaccine groups showed similar response profiles. Antibody-binding titers to H1N1pdm09 and virus-neutralizing responses to A/swine/Iowa/15/30 (H1N1) and NJ/8/76 strains were similar between the two vaccine groups, suggesting that monovalent immunization with the most recent H1N1 strain (Bris/59/07) was sufficient to generate this level of broadening of immune recognition without using a multivalent vaccine strategy. Notably, vaccination with the A/New Jersey/1976 swine influenza vaccine can induce HA-stem and cross-neutralizing responses to H1N1pdm09 in humans (60), supporting cross-recognition between these strains as demonstrated in the present study. These cross-neutralizing responses were not observed in the control group, indicating that exposure to H1N1 strains and H1N1pdm09 are both required to achieve this breadth of recognition.

Contrary to the concept of OAS, in which subsequent infections with variant strains preferentially boost antibody responses to the original strain, we observed no increases in virus neutralization or HAI titers against four of the five H1N1 vaccine strains after H1N1pdm09 infection. Preferential boosting of primary responses has been recorded for both H3N2 and H1N1 subtype viruses in human and animal models (6–11, 61–64). However, it is unclear whether back-boosting also occurs after exposure to a highly divergent influenza virus of the same subtype, such as H1N1pdm09 in individuals previously exposed to H1N1. Our results indicate that antigenic distance between virus strains may limit the extent of back-boosting. In humans, assessments of back-boosting may be partly confounded by the lack of information regarding previous exposure history. For instance, previous exposure to 1976 swine influenza vaccine can influence responses to both H1N1pdm09 and H1N1 (33, 34). Although previous studies in influenza-naive animal models supported the concept of OAS, those studies were largely restricted to H1N1 viruses spanning a more limited period of virus evolution (1931 to 1956) (6, 7, 9). Additionally, studies in humans have generally been restricted to the pre-pandemic period of exposure to H1N1 viruses (10).

Animals were not euthanized at the end of the study. Therefore, it was not possible to study possible signs of vaccine-mediated enhancement of pathology. However, virus was completely cleared by day 14 after infection and it is unlikely that any remaining pathology could have been detected. Instead, animals were returned to the experimental stock for re-use or returned to the breeding colony.

In conclusion, the present study showed that, for H1N1 influenza viruses, a multivalent vaccine approach involving different H1N1 strains has limited potential in terms of generating cross-protective immune responses to highly divergent strains; multivalent vaccination only resulted in the accelerated induction of cross-neutralizing and HA-stem responses. Furthermore, back-boosting in a non-human primate model is limited to influenza virus strains of intermediate antigenic distance between the H1N1 vaccine and H1N1pdm09 challenge strain, indicating that OAS is influenced by viral divergence. Thus, OAS may be less restrictive than previously suspected, consistent with the reported induction of novel effective responses after exposure to highly heterologous influenza virus antigens (16, 20, 65).

## Data availability statement

The raw data supporting the conclusions of this article will be made available by the authors, without undue reservation.

## Ethics statement

The animal study was approved by Dierexperimentencommissie BPRC (DEC-BPRC). The study was conducted in accordance with the local legislation and institutional requirements.

## Author contributions

PM: Conceptualization, Formal Analysis, Investigation, Methodology, Project administration, Supervision, Writing – review & editing. DM: Writing – review & editing, Formal Analysis, Investigation, Methodology. AA: Formal Analysis, Investigation, Methodology, Writing – review & editing, Resources. AM: Writing – review & editing, Resources, Validation. RC: Resources, Validation, Writing – review & editing, Methodology. AR: Methodology, Resources, Validation, Writing – review & editing, Conceptualization, Supervision. PA: Conceptualization, Resources, Validation, Writing – review & editing, Supervision. ZF: Writing – review & editing, Investigation, Methodology. DE: Methodology, Writing – review & editing, Resources. NS: Funding acquisition, Writing – review & editing. OE: Funding acquisition, Resources, Writing – review & editing. EV: Supervision, Writing – review & editing. MG: Investigation, Methodology, Writing – review & editing. WB: Supervision, Writing – review & editing. MC: Conceptualization, Funding acquisition, Methodology, Resources, Supervision, Writing – review & editing, ER: Conceptualization, Funding acquisition, Writing – review & editing. GK: Writing – review & editing, Conceptualization, Funding acquisition, Project administration, Supervision, Writing – original draft.

## Supplementary material

Supplementary Figure 1 Antibody responses to HA proteins of all five vaccine strains and the challenge strain over time. IgG antibody responses over time against HA proteins of PR/8/34, USSR/92/77, Texas/36/91, NC/20/99, Bris/59/07, and Cal/07/09 are shown for each individual animal in the monovalent vaccine group (M1-M6), pentavalent vaccine group (P1-P6), and naive control group (C1-C4). Antibody levels are expressed as arbitrary units (AU), defined as the dilution where the OD_450_ value is 1 unit above background. Vertical dotted lines in graphs indicate time points of immunization; vertical dashed line indicates time of challenge.

Supplementary Figure 2 HAI responses to HA. HAI titers measured 3 weeks after the fourth immunization procedure and 56 days after challenge using the H1N1 vaccine strains PR/8/34 and Bris/59/07 and the H1N1pdm09 challenge strain Cal/04/09 among animals in the monovalent vaccine group (black circles), pentavalent vaccine group (black squares), and naive control group (white triangles). The lowest dilution tested was 1:10. Significant differences between groups were determined using the Mann–Whitney test. *p<0.05.
