# Supplementary figures and images for: Vaccine-induced neutralizing antibody responses to seasonal influenza virus H1N1 strains are not enhanced during subsequent pandemic H1N1 infection
Source: Front Immunol. 2023 Aug 24;14:1256094. doi: 10.3389/fimmu.2023.1256094 (PMC10484506; doi:10.3389/fimmu.2023.1256094)

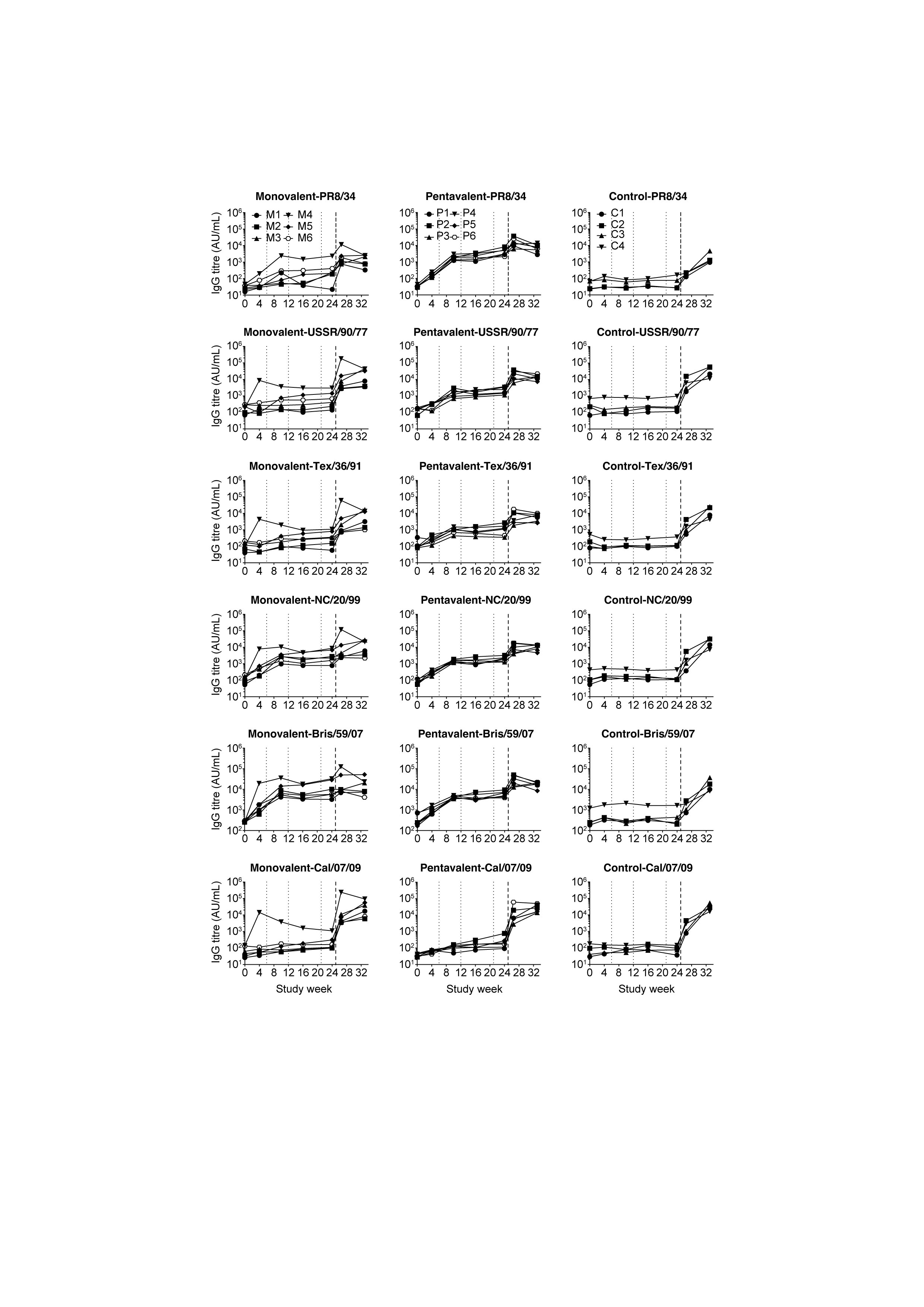

Supplement: Supplementary Figure 1 — Antibody responses to HA proteins of all five vaccine strains and the challenge strain over time. IgG antibody responses over time against HA proteins of PR/8/34, USSR/92/77, Texas/36/91, NC/20/99, Bris/59/07, and Cal/07/09 are shown for each individual animal in the monovalent vaccine group (M1-M6), pentavalent vaccine group (P1-P6), and naive control group (C1-C4). Antibody levels are expressed as arbitrary units (AU), defined as the dilution where the OD450 value is 1 unit above background. Vertical dotted lines in graphs indicate time points of immunization; vertical dashed line indicates time of challenge. [file Image_1.tif]

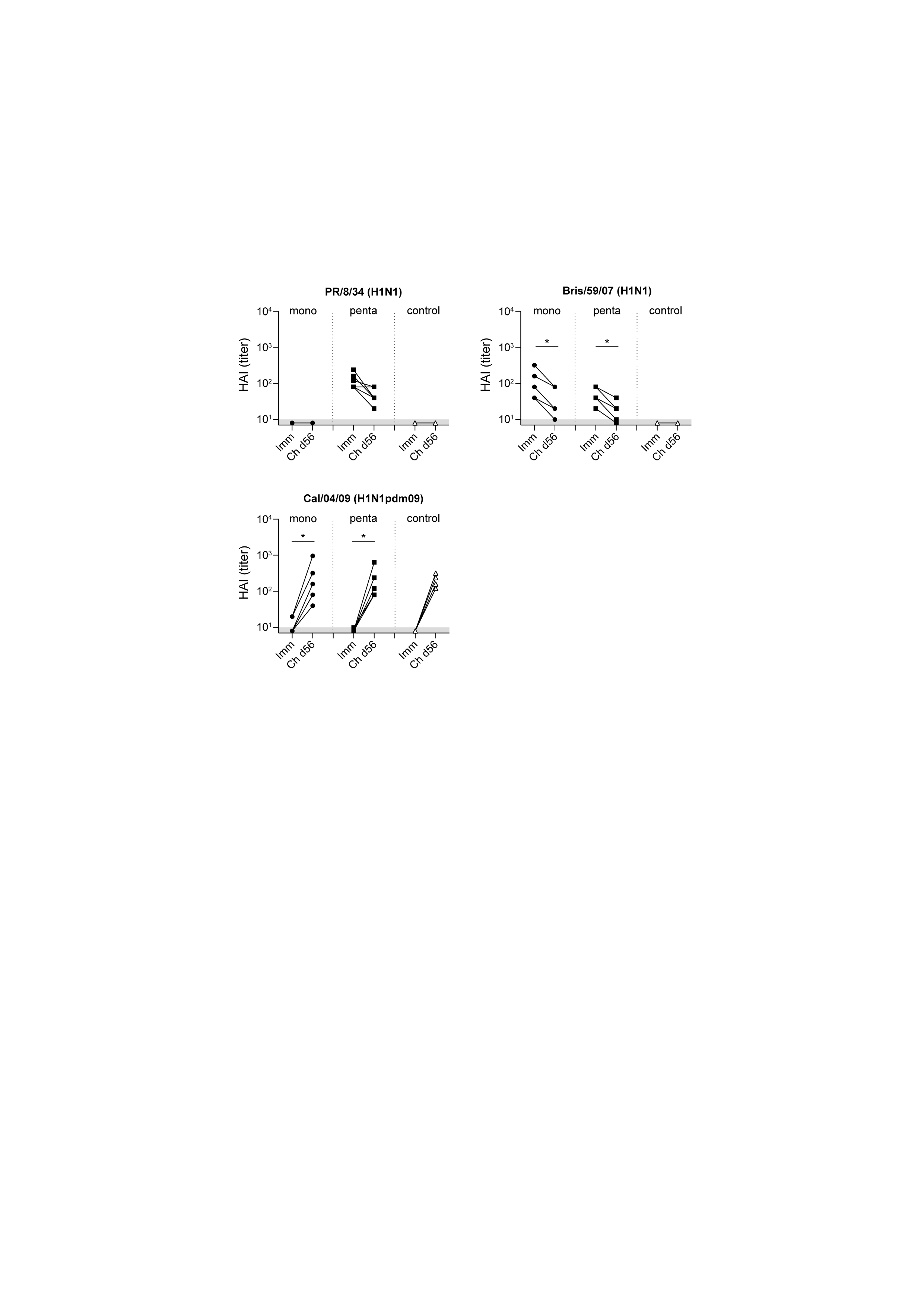

Supplement: Supplementary Figure 2 — HAI responses to HA. HAI titers measured 3 weeks after the fourth immunization procedure and 56 days after challenge using the H1N1 vaccine strains PR/8/34 and Bris/59/07 and the H1N1pdm09 challenge strain Cal/04/09 among animals in the monovalent vaccine group (black circles), pentavalent vaccine group (black squares), and naive control group (white triangles). The lowest dilution tested was 1:10. Significant differences between groups were determined using the Mann–Whitney test. *p<0.05. [file Image_2.tif]
