# Supplementary material for: Vaccine-induced neutralizing antibody responses to seasonal influenza virus H1N1 strains are not enhanced during subsequent pandemic H1N1 infection
Source: Front Immunol. 2023 Aug 24;14:1256094. doi: 10.3389/fimmu.2023.1256094 (PMC10484506; doi:10.3389/fimmu.2023.1256094)
Supplement: Supplementary file 3 [file Table_1.docx]

Table S1. Age and weight of animals from the study

| Group | Animal | Birth date | Age (months)* | Weight |
| --- | --- | --- | --- | --- |
| monovalent | M1 | 04-10-2010 | 72 | 8,62 |
| monovalent | M2 | 24-06-2009 | 88 | 7,70 |
| monovalent | M3 | 03-10-2009 | 84 | 7,72 |
| monovalent | M4 | 24-10-2009 | 84 | 7,04 |
| monovalent | M5 | 07-11-2009 | 83 | 7,04 |
| monovalent | M6 | 24-12-2009 | 82 | 7,42 |
|  |  | average | 82 | 7,59 |
|  |  |  |  |  |
| pentavalent | P1 | 11-02-2011 | 67 | 7,57 |
| pentavalent | P2 | 26-12-2011 | 58 | 5,65 |
| pentavalent | P3 | 14-09-2009 | 85 | 7,41 |
| pentavalent | P4 | 28-10-2009 | 84 | 8,97 |
| pentavalent | P5 | 02-10-2009 | 84 | 7,44 |
| pentavalent | P6 | 25-11-2009 | 83 | 8,59 |
|  |  | average | 77 | 7,61 |
|  |  |  |  |  |
| control | C1 | 30-04-2011 | 65 | 6,78 |
| control | C2 | 17-11-2009 | 83 | 6,67 |
| control | C3 | 21-12-2009 | 82 | 8,44 |
| control | C4 | 25-12-2009 | 82 | 7,56 |
|  |  | average | 78 | 7,36 |

*Age in months at time of first immunization procedure.
